# Evaluating urban oriental plane wood as a tonewood: effects of solvent extraction on acoustic performance

**DOI:** 10.1038/s41598-026-54185-w

**Published:** 2026-05-22

**Authors:** Mehran Roohnia

**Affiliations:** Handcraft Arts Association, Aziziye Mah. Hünkar Sk. No:30/A Meram, Konya, 42000 Türkiye

**Keywords:** *Platanus orientalis*, Tonewood, Solvent extraction, Damping capacity, Acoustic radiation coefficient, Musical instrument manufacturing, Engineering, Materials science

## Abstract

This research investigates the acoustic potential of mature Oriental plane wood (*Platanus orientalis* L.) harvested from urban environments for use in musical instrument manufacturing. While traditionally undervalued as low grade raw material, this species offers unique aesthetic properties that could be enhanced for high value lutherie applications. The study aimed to determine if solvent extraction could improve the wood’s acoustic properties to meet standard criteria for instrument soundboxes. Forty clear, straight grained radial specimens were prepared and subjected to sequential extraction using pure water and a 1:3 acetone-ethanol solution via a Soxhlet system. Dynamic mechanical properties were evaluated using a non-contact forced vibration system. Results indicated that while solvent washing consistently reduced air-dry density and the longitudinal modulus of elasticity, the acoustic radiation coefficient remained stable or slightly increased, particularly with the ethanol-acetone treatment. However, extraction significantly increased the damping capacity, suggesting that the removed extractives, much like those in Pernambuco wood (*Caesalpinia echinata* Lam.), play a structural reinforcement role within the cell wall. While plane wood requires careful impedance matching for use in resonance boxes, its extractives present a promising opportunity for impregnating other species to enhance their acoustic performance.

## Introduction

The term ‘tonewood’ denotes specific timber species selected for their superior vibro-acoustic performance, characterized by their capacity to efficiently radiate acoustic energy or sustain mechanical vibrations within musical instruments. As outlined by Bucur^1^, the selection of these materials is not merely traditional but is grounded in the wood’s ability to act as a mechanical filter and amplifier of string energy.

Traditionally, the choice of species is divided by functional role within the instrument. Softwood species such as Norway Spruce (Picea abies) and Sitka Spruce (*Picea sitchensis*) are the standard for soundboards. These species are prized for their high acoustic radiation. Hardwood species like Maple (*Acer sp.*) are typically utilized for the back and sides of instruments^1^. Thus, the selection of tonewood shifts from traditional craftsmanship to a systematic scientific application. This ensures a direct correlation between the mechanical properties of the timber and the resulting acoustic output, as will be discussed in this study.

The Oriental Plane tree (*Platanus orientalis* L.), which is native to the East Mediterranean region^2^ has long been recognized as a primary icon of urban green spaces. Due to its remarkable resilience against air pollution and its expansive canopy, this species has flourished for decades—and in many cases, centuries—along major city thoroughfares, reaching substantial diameters^3^. However, urban forest management frequently faces the challenge of aging trees that are either felled for infrastructure development or collapse due to natural factors such as storms and decay.

Currently, due to a lack of strategic high value utilization, the timber from these fallen trees might be swiftly removed and treated as low grade raw material for the particleboard, MDF (Medium Density Fiberboard) industries and fırewood. This practice effectively undermines the inherent value of the wood, particularly its unique “lacewood” (medullary ray) grain patterns and aesthetic texture. Such an approach not only results in the loss of a valuable natural resource but also overlooks its significant economic and artistic potential. In this context, utilizing *P. orientalis* wood for musical instrument manufacturing offers a sophisticated solution to increase value-added benefits. This industry utilizes both the acoustic and visual properties of the wood, breathing new life into the remains of ancient urban trees through the medium of art.

The internal regions of the log, specifically the juvenile wood surrounding the pith, are characterized by immaturity and structural vulnerability, rendering them unsuitable for the rigorous requirements of musical instrument manufacturing. Similarly, the outermost layers, or sapwood, are rich in nutrients and moisture, making them highly susceptible to biological degradation and physical instability. Consequently, only the mature heartwood—which constitutes a specific portion of the trunk—possesses the necessary density and stability for Lutherie^4^. This necessitates the use of large diameter, ancient trees. Fortunately, the felled plane trees (*Platanus orientalis*) from urban landscapes provide an abundant source of such mature, large diameter timber, offering a unique opportunity to reclaim high quality material that would otherwise be undervalued.

Furthermore, the mature heartwood must possess distinct acoustic properties to be considered viable for use in particular components of various musical instruments. Wegst^5^ established a comprehensive set of parameters to evaluate the acoustic performance of various tonewoods—research that was further highlighted and expanded upon by Bucur^6^ and later by Roohnia^7^. According to the Wegst criteria, wood suitable for the back and side of stringed instrument resonators should ideally exhibit a damping capacity between 0.008 and 0.018 and an acoustic radiation coefficient between 5 and 8 m^4^/kg.s.

Building upon these applications, our research group has spent the last decade investigating the influence of solvent washing and the reduction of extractive content on the acoustic properties of various wood species. Our previous findings have consistently demonstrated the potential of this approach. For instance, initial studies focused on the pure water washing processes of mulberry^8^ and maple wood (pure water and an ethanol-acetone mixture)^9^. Subsequent research expanded this scope to include species such as the Persian silk tree^10^. More recently, our team has integrated biological pre-treatments to optimize the process; Daeepour et al. and Zamaninasab et al. utilized baker’s yeast to facilitate extractive removal in pine and walnut wood, respectively^11,12^. The present study continues this established research trajectory, aiming to evaluate the efficacy of solvent washing on the acoustic performance of *Platanus orientalis*.

In addition to our work, researchers worldwide have explored this area and reported valuable results. To name a few, the effects of extraction treatment with deionized water, dichloromethane, benzyl alcohol, and ethanol on the acoustic vibrational properties of spruce wood were evaluated by Miao^13^. The degree of improvement in the acoustic vibrational properties of wood differed depending on the solvent used. Greater improvement was observed after extraction with benzoyl alcohol and dichloromethane as compared to ethanol and deionized water. Bremaud et al. studied the effect of extractives on the vibrational properties of African Padauk^14^. Extractions increased the damping capacity as much as 60%, irrespective of minute moisture changes or of initial properties. Apparent density was barely changed but, after correcting the mass contribution of extracts, was in fact slightly reduced. Se Golpayegani et al. studied the effect of extractions on the dynamic mechanical properties of white mulberry^15^. Removal of extractives caused the damping capacity to increase and the moduli to decrease. Acetone, the most effective solvent for damping despite a moderate extraction yield, increased the damping capacity by at least 20% but did not modify the specific stiffness as much. The changes in the acoustic properties of Western red cedar due to Methanol Extraction were studied by Yano^16^. Due to methanol extraction, the values of the damping capacity of heartwood specimens increased with a concomitant slight decrease in specific stiffness, while those of sapwood specimens decreased with a slight increase in specific stiffness. It was concluded that the damping capacity of the heartwood of Western red cedar is strongly affected by the methanol extractives existing in cell walls. In principle, reducing the content of extractives—which typically do not play a structural role in wood—is expected to maintain stiffness while decreasing damping capacity. However, in certain cases, such as in Pernambuco wood (*Caesalpinia echinata* Lam.), it has been observed that the removal or reduction of extractives leads to a significant increase in damping capacity. This suggests that such extractives play a beneficial role within the cell wall, which is weakened upon their removal^17–19^. Matsunaga et al. applied this concept in their research by extracting water soluble components from Pernambuco and impregnating spruce wood with them. The damping capacity of the impregnated specimens decreased—to nearly half of the original value—as weight gain increased. This suggests that the decrease in damping capacity results from the impregnation of extractive components into the amorphous regions of the cell walls, where they form secondary bonds between matrix substances^19^. Their targeted research, highlights the necessity of conducting similar studies on all wood species with the potential for use in the musical instrument industry. While some wood types may be unsuitable in their natural state, comprehensive knowledge regarding the effect of extractives on acoustic properties allows these available species to be treated, effectively serving as viable alternatives to rare or endangered wood samples. Therefore, beyond the objectives mentioned above, focusing this present research on the solvent washing of plane wood contributes significantly to a more comprehensive understanding of how extractives affect wood’s acoustic performance.

A review of the physical and mechanical properties of this species, alongside its extractive content as documented in the literature, provides essential context for this study. Previous researches indicates that the extractive content of oriental plane wood typically ranges between 2.5% and 4%^20^. GC-MS results identified 17 specific compounds in the oriental Plane wood. Hexadecanoic acid (22.18%), heptadecan-8-carbonic acid (15.24%), and 1,2-benzenedicarboxylic acid (12.08%) were found to be the major components^20^.

The oven-dry density of this species ranges from 0.5 to 0.7 g/cm³, categorizing it as a medium-density wood^21^. Furthermore, oriental plane wood has a relatively high volumetric shrinkage of about 13% to 14%^22^. The modulus of elasticity for this wood is generally reported to be between 5 and 7 GPa^21,22^.

This study investigates the potential of mature Oriental plane wood (*Platanus orientalis*) for use in musical instrument soundboxes. Specifically, it examines whether solvent extraction and the subsequent reduction of extractive content can enhance the wood’s properties, thereby justifying its use as a tonewood. The primary objectives of this study are defined as follows:

- To transition the use of urban plane tree wood from low value material to high value aesthetic applications through specialized reclamation.
- To integrate plane wood into existing wood science research by documenting its specific properties.
- To investigate how solvent washing and the reduction of extractive content influence the acoustic properties of various wood species.

## Experimental section

### Materials

A broken trunk from a street plane tree in the Karaj region - Iran, with a diameter of 80 cm, was transported to the laboratory of the Darkoob Non Destructive Testing Knowledge Based Group. Forty clear, straight grained radial specimens were prepared from the healthy mature wood in the heartwood section. Sample dimensions were 140 × 14.5 × 2 mm (L×R×T). These samples were cut parallel to the fiber direction along the longitudinal axis and were numbered for identification. A laboratory bench scale with an accuracy of 0.01 g was used to measure sample mass, while a vernier caliper with a precision of 0.02 mm was used for dimensional measurements.

To reach equilibrium, the samples were stored for three weeks at 20 ± 1 °C and 65 ± 5% relative humidity (ISO 3129 − 2019)^23^. This acclimatization process, starting from the green state, ensured that dimensional shrinkage and swelling had stabilized. Under these conditions, the nominal moisture content (MC) of the wood is approximately 12%, though it may vary slightly based on the physic-chemical composition of each sample. To maintain stable environmental conditions for the specimens, a constant climate chamber (KBF series, BINDER GmbH, Tuttlingen, Germany) was employed.

While precise measurement of the equilibrium moisture content (EMC) typically requires oven drying to 0% MC, this was intentionally avoided. Such extreme drying can cause thermal shock and damage the wood cell walls. Therefore, to preserve the integrity of the samples and avoid errors caused by repeated drying and wetting cycles, the nominal EMC was used for the study. In parallel, three samples—identical to the test pieces—were used to monitor the equilibrium moisture content at each step. These reference samples were freely dried to an oven dry state to verify the results. In any case, given the significant impact of moisture content on density, modulus of elasticity^24^, and their derived parameters, even an approximate estimate is far more valuable than no information at all.

### Method

These forty samples were divided into two groups of twenty. One group was initially extracted with pure water, while the other was treated with an acetone-ethanol solution in a 1:3 ratio (v/v), (Merck; Darmstadt, Germany) (purity: acetone, 99.5% - ethanol, 96%). The extraction was performed using a conventional glass Soxhlet apparatus for eight hours—a duration chosen due to the thinness of the samples. Subsequently, the treatments were reversed: the water washed group was treated with acetone-ethanol, and the acetone-ethanol group was treated with pure water. After each stage, the samples were re-conditioned under the previously mentioned temperature and humidity settings before undergoing acoustic vibration testing.

**Fig. 1:**
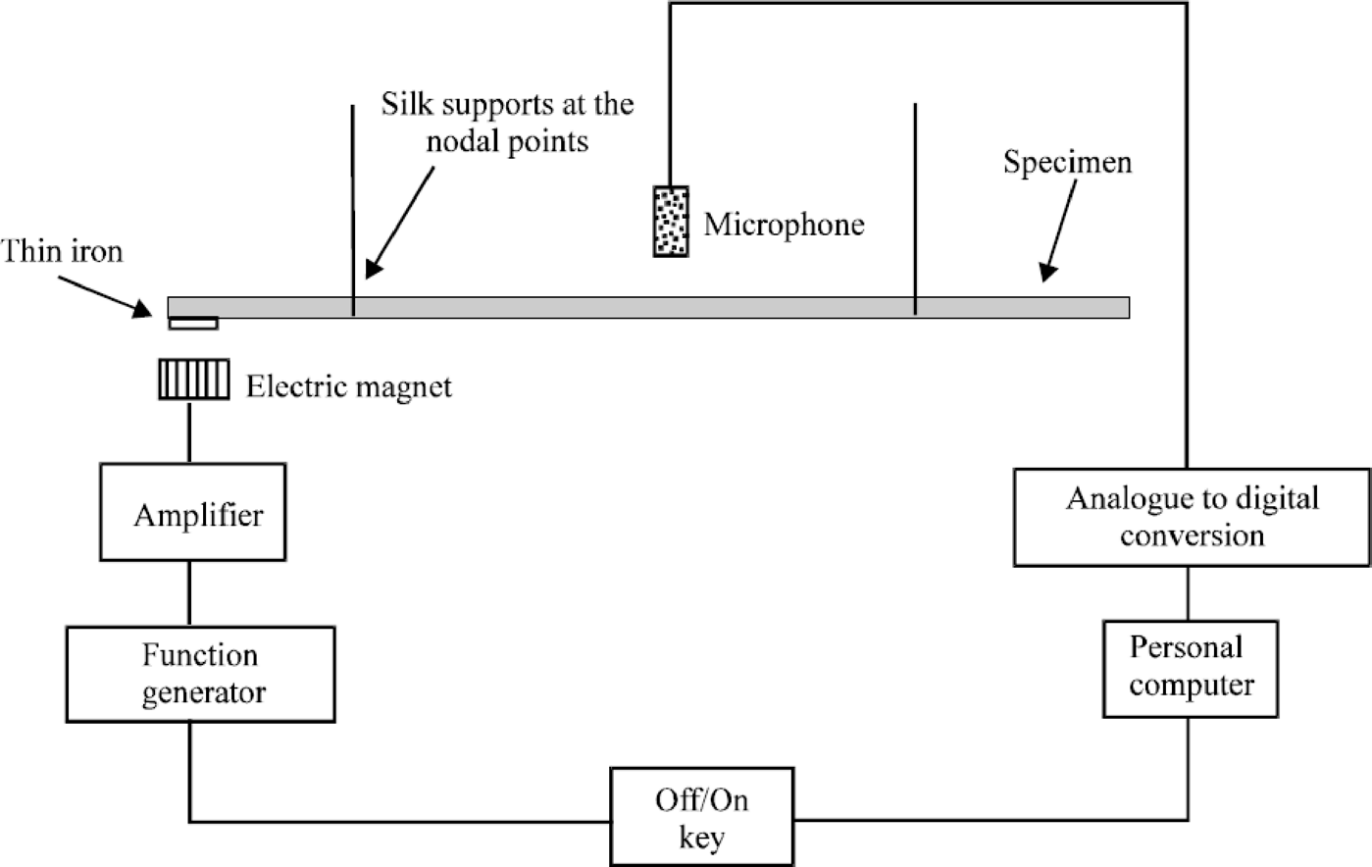
Schematic setup for contactless forced vibration test^25^.

Under equilibrium conditions, apparent air-dry density was determined by the mass to volume ratio of the samples. To estimate the longitudinal dynamic modulus of elasticity (*E*_*d*_) along the grain, through the Euler-Bernoulli theory, Eq. (1), a non-contact forced vibration system was employed using a free-free beam configuration (Fig. 1). This apparatus, already developed and calibrated by the author through comparison with static bending tests and standard vibro-acoustic systems, has been extensively validated in previous research^9–12,25^.

1 $${E_d}=\frac{{4{\pi ^2}{f^2}{L^4}A\rho }}{{{{4.73}^4} \times I}}$$

where; *f* is natural flexural frequency (*Hz*); *L* is length of the specimen (*m*); *A* is cross section area (*m*^*2*^); *ρ* is the density (*Kg/m*^*3*^), and *I* is the moment of inertia of the cross section (*m*^*4*^). The constant 4.73 corresponds to a bar with free-free boundary conditions vibrating in its fundamental flexural mode.

The Acoustic Characteristic Impedance (*z*) and the Acoustic Radiation Coefficient (*K*) are calculated from the density and modulus of elasticity, as shown in Eqs. (2) and (3). While *z* is a fundamental parameter governing energy transmission efficiency through impedance matching at boundaries, *K* serves as a decisive factor in determining the overall sound radiation and perceived loudness of a musical instrument^7^.

The Acoustic Characteristic Impedance (*z*) and Acoustic Radiation Coefficient (*K*), i.e. two critical acoustic parameters, are then calculated from the density and modulus of elasticity as shown in Eqs. (2) and (3)^7^.

2 $$z = \sqrt {{E_d}\cdot \rho }$$

3 $$K = \sqrt {\frac{{{E_d}}}{{\rho {}^3}}}$$

**Fig. 2:**
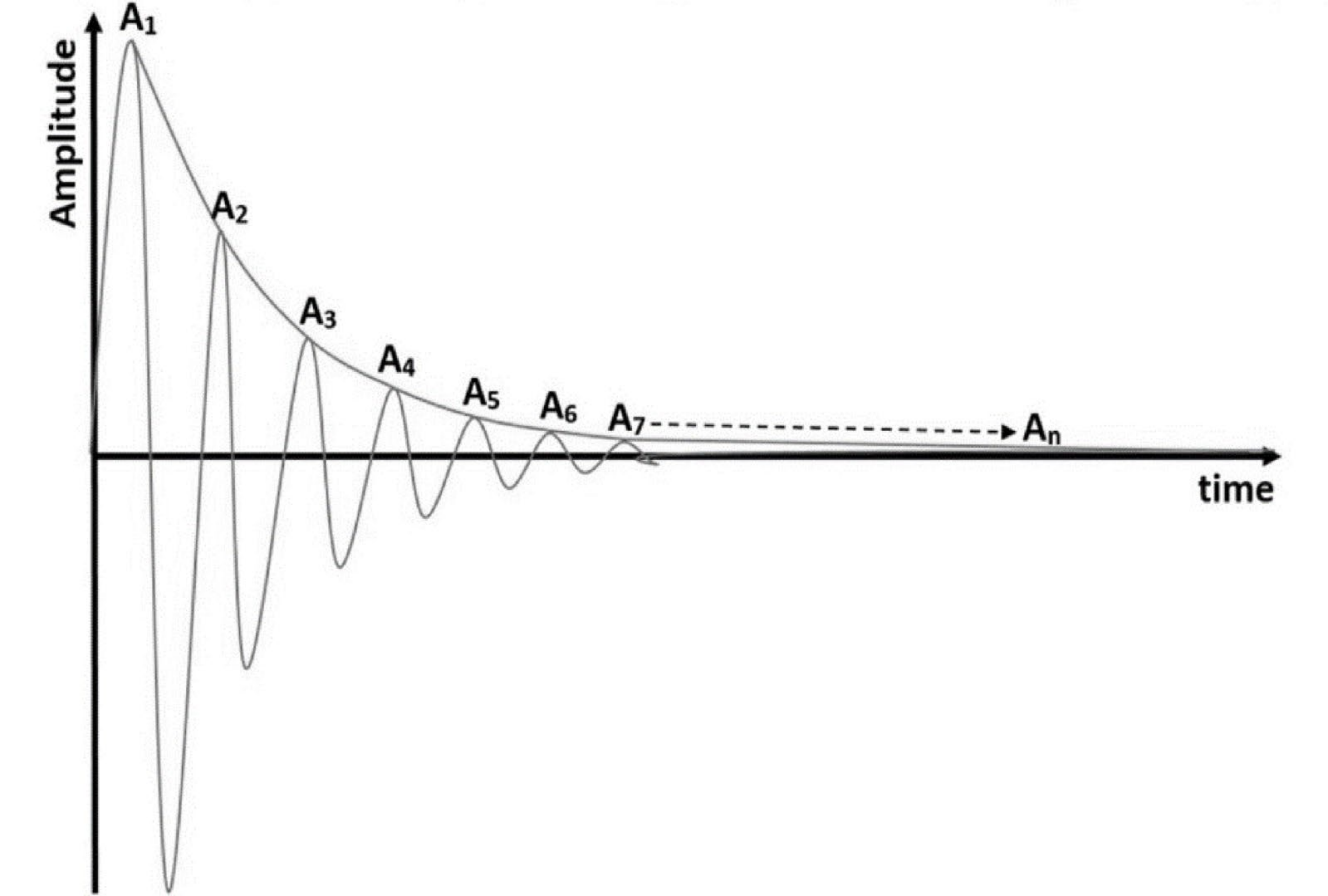
Damping in logarithmic scale^7^.

Damping capacity (tanẟ) of the specimens was determined using logarithmic decrements of the vibration in Eq. (4), immediately after stopping the driving force (Fig. 2).

4 $$\tan \delta = \frac{1}{{n\pi }}\ln \frac{{{A_i}}}{{{A_{i + n}}}}$$

Audacity software was used for audio collection; the saved files were then exported to NDT-Lab software^25^ for processing.

To evaluate the alterations in the studied acoustic parameters, linear regression analysis was employed. The relationship between the acoustic properties before and after the washing treatment was modeled, and the goodness of fit was assessed using the coefficient of determination (*R*^*2*^). To ensure the statistical reliability of the observed trends, a significance test was performed for the regression model, and the results were reported as p-values. A p-value of less than 0.05 was considered statistically significant.

To maintain conciseness, graphs from similar categories were consolidated. Due to the proximity of the regression lines, distinct dotted and dashed patterns were employed for differentiation. For enhanced clarity, each regression equation and its corresponding *R*^*2*^ value were enclosed in a box formatted with a line pattern matching its respective regression line.

## Results and discussion

First, the equilibrium moisture content (EMC) of the control samples (*n* = 3) before (raw) and after the extraction rounds is reported in Table 1. As a reminder, the first group was washed with pure water in the first round, followed by an ethanol-acetone solution in the second. The second group was washed with the ethanol-acetone solution first, followed by pure water in the second round.

**Table 1:** The equilibrium moisture of control samples (*n* = 3) before and after solvent extraction.

	Group1		Group2	
	MC%	Standard deviation	MC%	Standard deviation
Raw	10.0	0.1	10.1	0.1
1st round	11.1	0.1	11.0	0.2
2nd round	11.4	0.1	11.1	0.1

**Fig. 3:**
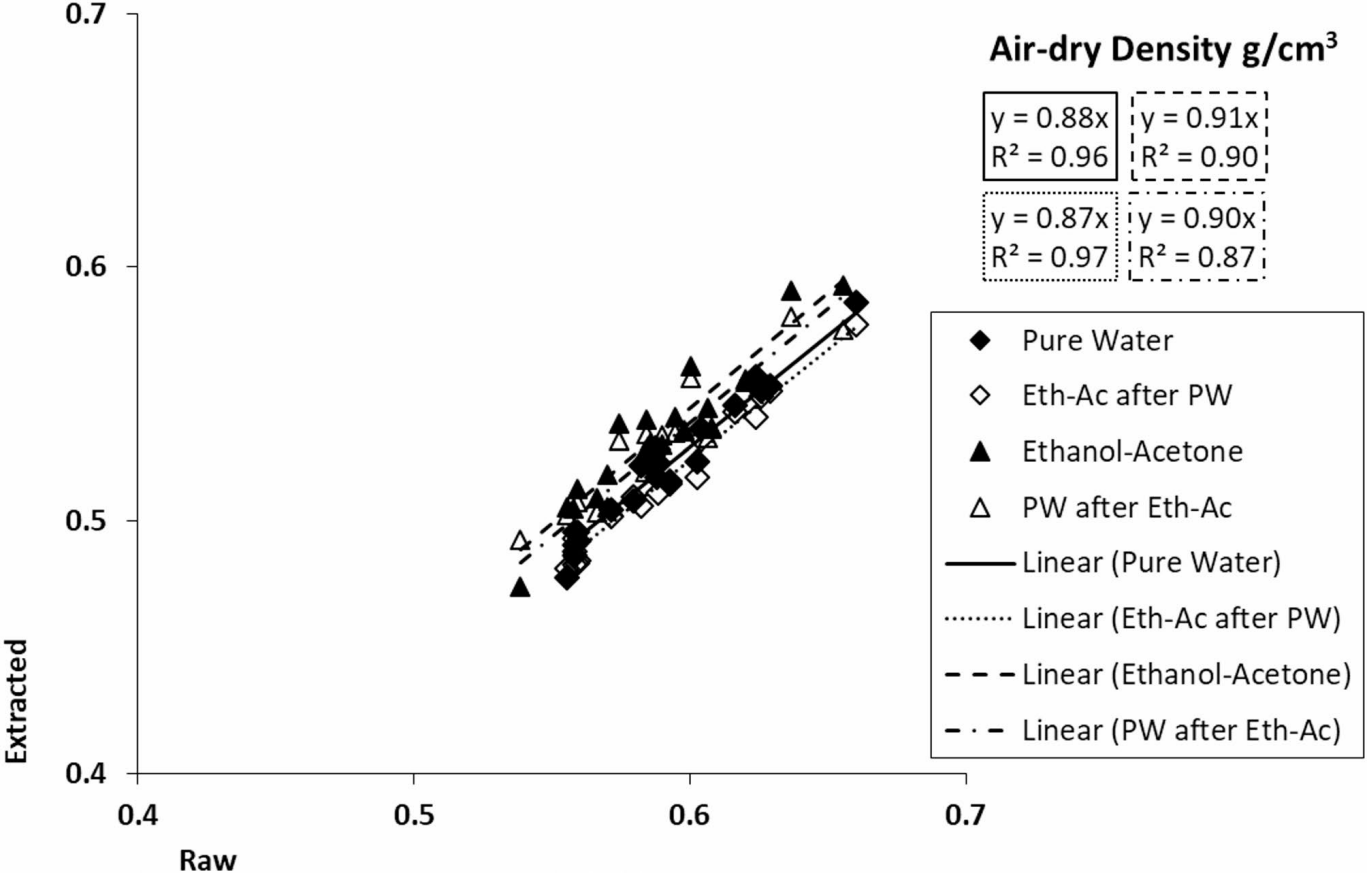
Correlation between the raw and extracted air-dry density (g/cm^3^) of the specimens following different solvent washing treatments. The plot compares the efficacy of Pure Water (PW), Ethanol-Acetone (Eth-Ac), and sequential washing steps (Eth-Ac after PW and PW after Eth-Ac). Linear regression lines indicate a strong positive correlation (R^2^ ≥ 0.87) across all treatments, with the Ethanol-Acetone treatment showing the highest extraction gradient (y = 0.91x). Based on 20 observations in each treatment, all treatments yielded a p-value < 0.01, confirming that the observed changes are statistically significant.

Subsequently, the air-dry density of the main samples (*n* = 20) was evaluated to assess the extent to which water and ethanol-acetone extraction removed soluble extractives and consequently reduced the sample density (Fig. 3).

The strong linear correlation (R^2^ ≥ 0.87) across all treatments indicates that the extraction of substances from our Platanus wood samples occurs proportionally to its initial air-dry density. The Ethanol-Acetone treatment exhibited the steepest slope (y = 0.91x) compared to that of the pure water, suggesting it is slightly less aggressive in density reduction compared to the sequential “Eth-Ac after PW” treatment (y = 0.87x). These results demonstrate that while solvent washing consistently reduces air-dry density, the magnitude of the effect is highly predictable.

**Fig. 4:**
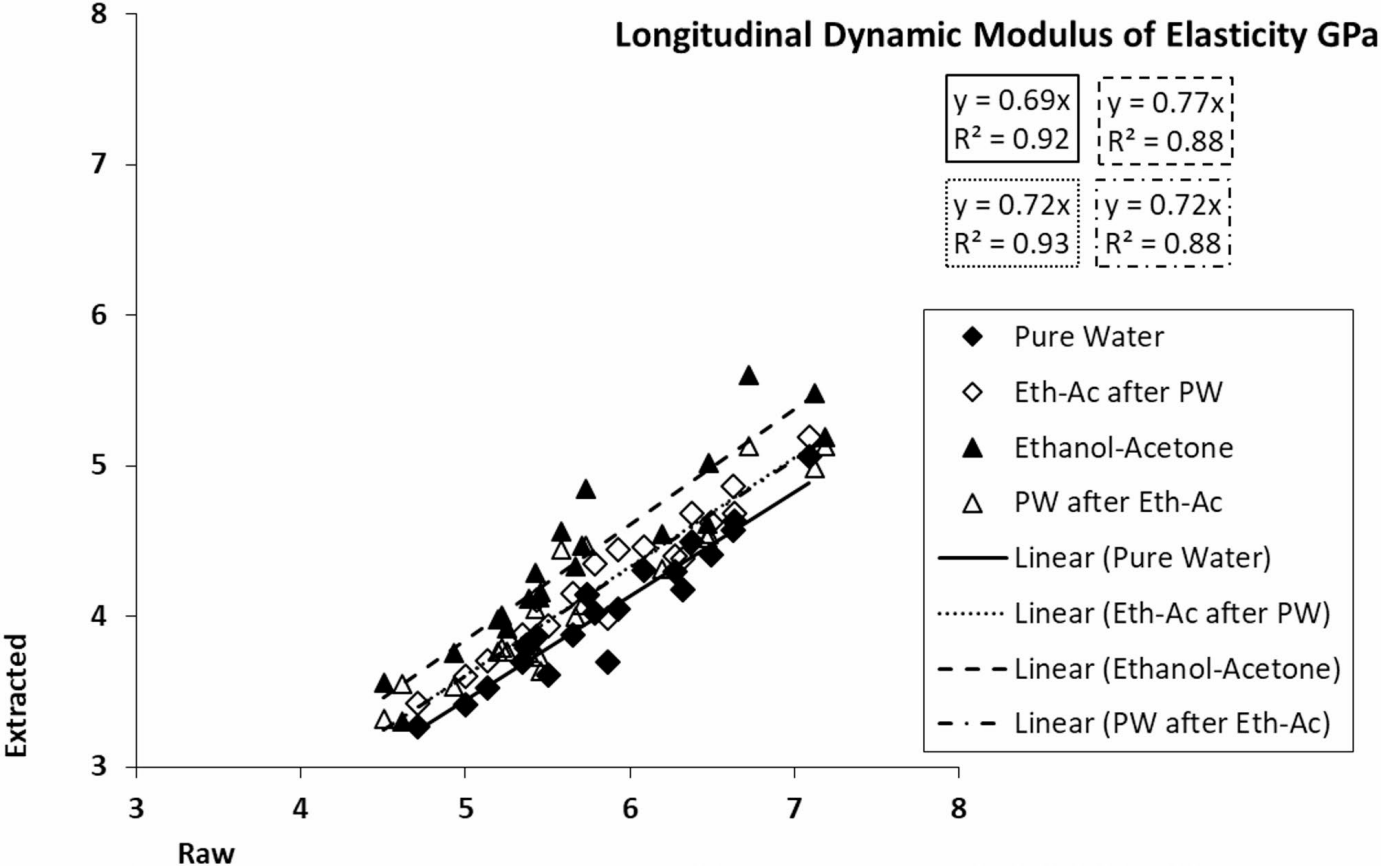
Relationship between the raw and extracted longitudinal dynamic modulus of elasticity (GPa) of Platanus wood samples subjected to different solvent washing protocols. The high coefficients of determination (R^2^ ≥ 0.88) indicate that the stiffness of the wood after extraction remains highly dependent on its initial elastic properties, with slopes ranging from 0.69 to 0.77. Based on 20 observations in each treatment, all treatments yielded a p-value < 0.01, confirming that the observed changes are statistically significant.

Another fundamental parameter in the vibrational behavior of wood is the dynamic modulus of elasticity along the grain. The following analysis examines how successive solvent extractions influenced this parameter (Fig. 4).

The extraction process results in a consistent reduction in the longitudinal dynamic modulus of elasticity, as evidenced by slopes significantly lower than unity (0.69 to 0.77). The Pure Water treatment appears to have the most pronounced impact on stiffness reduction (y = 0.69x), whereas the Ethanol-Acetone treatment preserves a higher proportion of the original dynamic moduli of elasticity (y = 0.77x). Despite these variations in solvent intensity, the strong linear correlations (R^2^ ≥ 0.88) suggest that the relative mechanical hierarchy of the wood samples is maintained throughout the extraction process.

At first glance, this decrease in the dynamic elastic modulus might be attributed to cell wall damage caused by successive drying and wetting cycles. While this is a valid hypothesis, it is important to note that the samples were thin, straight grain, and intact radial cuts. Furthermore, the conditioning process was carefully managed from the green state to the fiber saturation point. Therefore, such a significant drop in the dynamic elastic modulus likely results from the loss of extractives that previously provided structural reinforcement within the cell walls. Following an analysis of other acoustic parameters, a more comprehensive discussion of this phenomenon will be presented.

The decreases in the modulus of elasticity and density values directly affect the acoustic impedance and the acoustic radiation coefficient. These will be analyzed in sequence.

**Fig. 5:**
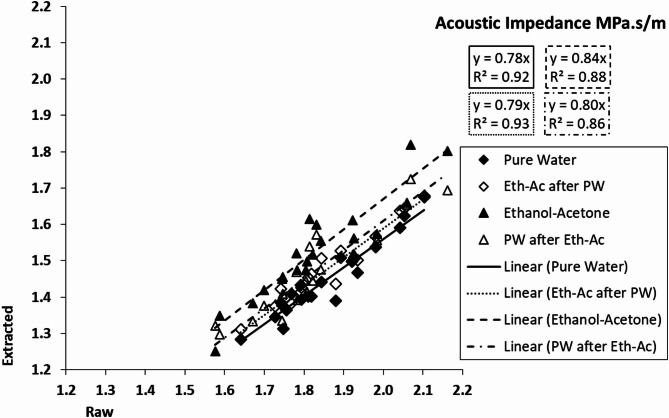
Correlation between the raw and extracted acoustic impedance (MPa s/m) of Platanus wood across four different solvent washing protocols. The linear regression models, all display high degrees of correlation (R^2^ ≥ 0.86). The slopes ranging from 0.78 to 0.84 quantify the reduction in impedance resulting from the removal of extractives, which alters both the density and sound velocity of the wood. Based on 20 observations in each treatment, all treatments yielded a p-value < 0.01, confirming that the observed changes are statistically significant.

As shown in Eq. (2), a relative decrease in density and elastic modulus leads to a predictable reduction in acoustic impedance (Fig. 5). While a lower impedance is generally desirable to reduce vibrational resistance, it is important to note that lowering the wood’s impedance can lead to an impedance mismatch with adjacent components under specific boundary conditions^7^.

Consequently, this factor must be carefully evaluated and adjusted based on the specific instrument type, as well as the material and impedance of the strings, bridge, and other structural components. When proper impedance matching is achieved, the expectation of enhanced vibration in the wood is technically sound.

The extraction process causes a systematic reduction in acoustic impedance, with the Ethanol-Acetone treatment showing the highest retention of the original impedance (y = 0.84x). Conversely, treatments involving Pure Water (either alone or as a primary step) resulted in lower slopes (0.78 to 0.79), indicating a more significant drop in impedance. The high R^2^ values suggest that the acoustic impedance of extracted wood remains highly predictable based on its initial state.

**Fig. 6:**
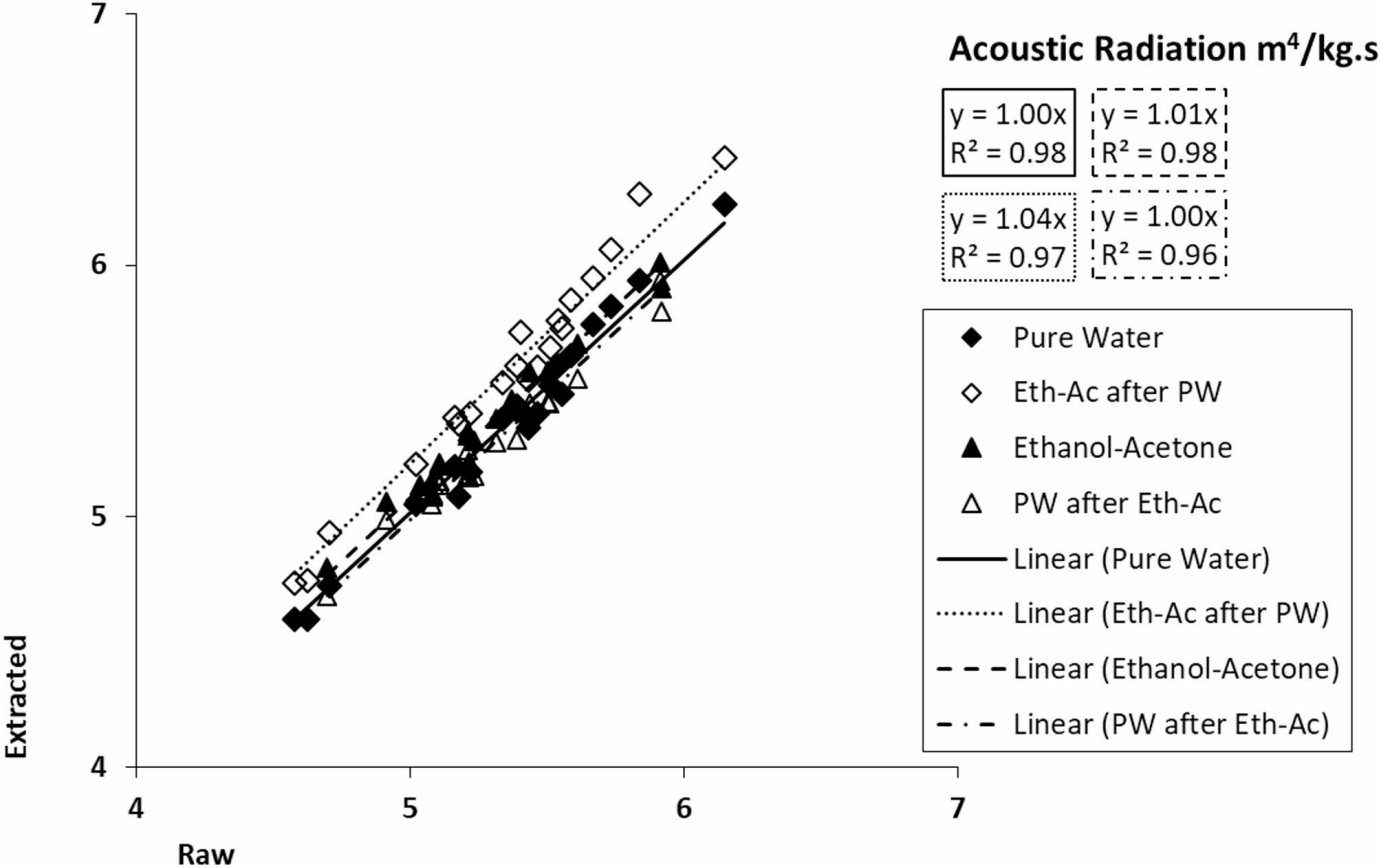
Correlation between the raw and extracted acoustic radiation coefficient (m^4^/kg s) of Platanus wood samples under various solvent washing conditions. The regression lines show exceptionally high linear agreement (R^2^ ≥ 0.96). Unlike other physical properties, the slopes near unity (1.00 to 1.04) indicate that the acoustic radiation coefficient remains largely stable or slightly increases following the extraction of wood solubles. Based on 20 observations in each treatment, all treatments yielded a p-value < 0.01, confirming that the observed changes are statistically significant.

To ascertain whether both the dynamic modulus of elasticity and density decreased, Eq. (3) must be employed to evaluate the fluctuations in the acoustic radiation coefficients following solvent washing (Fig. 6).

The extraction process appears to have a negligible or slightly positive effect on the acoustic radiation coefficient, with slopes ranging from 1.00 to 1.04. The “Eth-Ac after PW” treatment showed the most distinct increase (y = 1.04x), likely due to a greater relative reduction in air-dry density compared to the change in stiffness. These results suggest that solvent washing can enhance or maintain the sound radiation efficiency of Platanus wood, making it a potentially beneficial treatment for acoustic applications where high radiation is desired.

Consequently, using solvent-washed wood for the back panels of a resonance box offers a greater potential for acoustic activation than using untreated wood. However, considering the permissible acoustic radiation coefficients for backs and sides (between 5 and 8 m4/kg s) established in previous literature^7^, a few of the plane tree samples tested in this study were smaller than the allowed value. Solvent-washing treatments are clearly necessary to improve this parameter. Specifically, washing with an ethanol-acetone mixture yielded the best results in increasing the acoustic radiation coefficient of the samples under study.

**Fig. 7:**
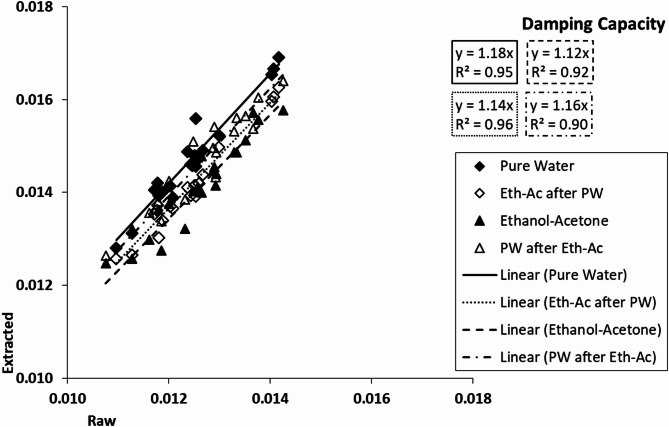
Relationship between the raw and extracted Platanus wood samples in terms of the damping capacities across different solvent washing protocols. The linear regressions show strong positive correlations (R^2^ ≥ 0.90). Slopes greater than unity (1.12 to 1.18) indicate that the extraction of solubles leads to a systematic increase in the damping capacity of the wood. Based on 20 observations in each treatment, all treatments yielded a p-value < 0.01, confirming that the observed changes are statistically significant.

The damping capacity results for both the raw and solvent washed samples fall within the permissible range of 0.008 to 0.018—a standard established by previous studies^7^ for the back and side panels of musical instrument resonator boxes. However, solvent washing significantly increased the damping capacity of the samples (Fig. 7). This suggests that extractives, particularly pure water soluble ones, play a structural strengthening role in plane wood; once these materials are removed, vibration retention is lost and damping increases. These findings align closely with the results reported by Matsunaga et al. for Pernambuco wood^18,19^. Furthermore, the potential success of impregnating other woods e.g. with the water soluble extractives of plane wood—similar to Matsunaga’s research on Pernambuco extractives and spruce wood^19^—remains a compelling area for future investigation.

A closer examination of this chart reveals that the solvent extraction process consistently increases the damping capacity of Platanus wood samples, as indicated by regression slopes ranging from 1.12 to 1.18. The Pure Water treatment resulted in the highest increase (y = 1.18x), while the Ethanol-Acetone treatment showed the most conservative change (y = 1.12x). These findings indicate that the removal of extractives likely eliminates substances that inhibit internal friction or modifies the cell wall matrix, thereby increasing the wood’s capacity for vibrational energy dissipation.

## Conclusions

Analysis of the sound radiation coefficient and damping capacity suggests that plane wood samples in this study are most suitable for the back and side plates of musical instrument resonance boxes. However, its acoustic impedance values fall below the standard threshold for these components. Plane wood wood samples in this study can be effectively utilized for these parts only if the other instrument components are adjusted to ensure proper impedance matching; otherwise, the acoustic waves will not reflect completely upon hitting the resonance box’s inner walls. The following specific conclusions were reached:

- In its natural state, plane wood does not possess the inherent properties required to meet the high performance acoustic objectives of resonance boxes.
- The application of solvent washing can marginally enhance the acoustic radiation coefficients of this species by up to 4%. Notably, solvent washing reduced the air-dry density of the tested wood by up to 13%.
- Solvent washing consistently increases damping capacity (by up to 18%). By correlating this increase with the observed reduction in elastic modulus (by up to 31%) and comparing to that of the Pernambuco wood, it is suggested that plane wood extractives might similarly play a structural reinforcement role within the cell wall.
- Structural analysis—specifically SEM, XRD, GC-MS and FTIR—alongside microstructural evidence, fully substantiates the role of extractives in strengthening cell walls. Future research by experts in these specialized fields is highly recommended to build upon these findings.
- Given these findings, further research is recommended into the impregnation of other wood species with plane wood extractives (specifically water soluble ones). This mirrors successful previous studies involving the injection of Pernambuco extractives into plane wood cell walls to reduce damping capacity. The prospects are promising, especially as plane wood extractives are easier to collect than Pernambuco. If successful, this method will likely become very popular.
- For future research, it is recommended to conduct experimental validation at the instrument level (e.g., soundboard testing and modal analysis of fully assembled resonance boxes).
- At an industrial level, this study provides an step forward for upgrading undervalued urban *P. orientalis* timber into higher value lutherie materials through predictable solvent treatments. The strong linear correlations observed allow manufacturers to standardize extraction protocols to achieve specific acoustic targets, such as enhanced radiation efficiency. Meanwhile, the potential to utilize Platanus extractives as bio-based acoustic enhancers for other species may offer a scalable and sustainable alternative to the use of rare or endangered tonewoods, provided that future research confirms these very initial findings.

## Data Availability

The data that support the findings of this study are available from the corresponding author upon reasonable request.
